# Site-Selective Controlled Dealloying Process of Gold-Silver Nanowire Array: a Simple Approach towards Long-Term Stability and Sensitivity Improvement of SERS Substrate

**DOI:** 10.1038/srep39115

**Published:** 2016-12-13

**Authors:** Natta Wiriyakun, Karuna Pankhlueab, Suwimon Boonrungsiman, Rawiwan Laocharoensuk

**Affiliations:** 1 National Nanotechnology Center (NANOTEC), National Science and Technology Development Agency (NSTDA), Pathum Thani 12120, Thailand

## Abstract

Limitations of achieving highly sensitive and stable surface-enhanced Raman scattering (SERS) substrate greatly concern the suitable method for fabrication of large-area plasmonic nanostructures. Herein we report a simple approach using template-based synthesis to create a highly ordered two-dimensional array of gold-silver alloy nanowires, followed by the controlled dealloying process. This particular step of mild acid etching (15%v/v nitric acid for 5 min) allowed the formation of Raman hot spots on the nanowire tips while maintaining the integrity of highly active alloy composition and rigid nanowire array structure. Full consideration of SERS substrate performance was accomplished using 4-mercaptobenzoic acid (4-MBA) as a probe molecule. Exceedingly higher SERS signal (150-fold) can be achieved with respect to typical gold film substrate. Moreover, an excellent stability of SERS substrate was also determined for over 3 months storage time. In contrast to the previous studies which stability improvement was accomplished at a cost of sensitivity reduction, the simultaneous improvement of sensitivity and stability makes the controlled dealloying process an excellent choice of SERS substrate fabrication. In addition, uniformity and reproducibility studies indicated satisfactory results with the acceptable values of relative standard deviation.

Surface-enhanced Raman spectroscopy is a well-known technique used for obtaining the information of molecules. It is widely recognized that when molecules are attached or located in the close proximity of surface-enhanced Raman scattering (SERS) surface, it leads to the amplification of the characteristic signal of the molecules in vibrational mode. Given that the strong enhancement factor (EF) of 10^5^–10^11^ has been reported previously, the signal of single molecule can be obtained^1^,^2^. In addition, successful measurements of SERS signal in the complicated matrix such as urine or blood have been demonstrated^3^. Therefore, SERS technique has been considered a powerful and versatile analytical tool for chemical and bio-sensing applications.

A SERS substrate is generally fabricated from metallic nanostructures, i.e. Au, Ag, and Cu. Both chemical and electromagnetic enhancement mechanisms are well accepted as key contributors to SERS phenomenon^4^. The latter is responsible to induce high field enhancement of the collective electronic oscillations on the metallic surface called surface plasmon resonance (SPR). The enhancement of SPR directly refers to the SERS sensitivity that mostly depends on various parameters of the metallic nanostructures such as dimension, morphology, composition, and shape. High field enhancement for SERS is typically generated at very specific area of metallic nanostructures and their aggregates called ‘hot spot’, which is located between the gap of nanoparticles aggregates or at the tip of anisotropic nanostructures such as nanorods and nanowires.

Nanowires (NWs) have received a great interest as building blocks for hierarchical assembly of complex nanostructures. Diverse elemental compositions and tailored physical properties (shape and size) of nanowires provide a great potential to utilize these structures in SERS-based sensing applications^5^. However, when considering the applicability of SERS substrate in real applications, not only sensitivity and reproducibility are crucially important, but stability and large-area uniformity are also strongly contributed to the successful implementation. Therefore, one should consider all 4 aspects (sensitivity, stability, uniformity and reproducibility) towards the development of SERS substrate.

In term of sensitivity, silver is considered the most useful for SERS enhancement. Most of the NWs-based SERS substrate fabrications have been carried out using either silver nanowires or silver-coated nanowire surfaces^6^,^7^,^8^,^9^,^10^. For example, a reproducible SERS-based platform capable of bacteria detection was reported using silver nanoparticles (Ag NPs)-decorated silicon nanowires^6^. A highly sensitive and reproducible SERS substrate was also fabricated by sputtering a silver layer onto the freestanding polymer nanopillars casted from anodized aluminum oxide (AAO) template^9^. Even though, the use of Ag NWs in SERS applications offers great electromagnetic field enhancement especially hierarchical Ag structures, the major drawback of metallic Ag is the rapid oxidation at the surface of nanostructures. This results in a dramatic decrease of SERS activity over time^11^,^12^.

Alternatively, gold nanowires (Au NWs) are promising candidate due to its biocompatibility and high stability. However, Au NWs generally provide lower SERS activity than silver^13^. To overcome the shortcoming of the monometallic nanowires as SERS substrates, some of the latest works successfully employed alloy nanowires as SERS substrates with high SERS enhancement and biocompatibility^13^,^14^. To further improve the SERS sensitivity, the construction of the porous metallic structure of these nanowires has been investigated to increase SERS hotspots. Several methods have been developed including galvanic displacement of Ag NWs with HAuCl_4_ solution to form bimetallic porous structure^15^, high temperature thermal evaporation to create porous Ag NWs^16^, and chemical/electrochemical dealloying of metal and multi-component alloy^17^,^18^. Among these methods, chemical dealloying has been widely used for achieving well-defined three-dimensional nanoporous structures due to simplicity of the process. Several examples of porous NWs structure are generally fabricated and used in the form of free-standing structures. For example, porous Au nanowires were obtained from dealloying of silver within Au-Ag NWs using concentrated HNO_3_ at 1 °C for 60 min^19^ or 35% HNO_3_ at room temperature for 15 min^20^. Highly porous silver-gold nanowires were fabricated by selective dissolution of silver from the free-standing Au-Ag alloy nanowires using 65% nitric acid for 3 hours^21^. Ag NWs with roughened surface can also be generated by chemical etching of Ag NWs using metallographic etchant (concentrated NH_4_OH and 30% H_2_O_2_)^17^. According to the previous reports, the dealloying process involved the use of highly concentrated acid (35–65% v/v). This leads to a significant change of Au-Ag alloy composition (higher atomic percentage of Au due to the dissolution of Ag); hence, a highly porous structure was obtained. Even though there is an advantage of high-density SERS hot spots within porous structure, the substantial Ag removal contrarily affects the SERS signal. In addition, SERS measurement is mostly accomplished using a single NW or an unorganized NWs structure. Thus, the major challenge lies in the implementation of such structure in practical applications, particularly considering the uniformity and simplicity of detection process. Moreover, the stability of these NW structures has not been studied.

On the other hand, research and development of a highly stable SERS substrate is focused on the use of air stable coating layer to protect the reactive surface of Ag from oxidation. Several designs of SERS structures were demonstrated using a thin oxide layer (i.e. TiO_2_, Al_2_O_3_)^11^,^22^, Au shell^23^, and graphene coatings^24^. Unfortunately, a long-term stability cannot be achieved without compromising SERS sensitivity. It has been reported in several studies that a thin layer (1–2 nm) of Al_2_O_3_ and TiO_2_ coating can cause approximately 50% reduction of SERS signal. Similar behavior was obtained for other coating materials because of the inaccessibility of Raman probes towards SERS active surface.

In order to achieve an ideal SERS substrate, this work focused on all critical aspects including sensitivity, stability, uniformity and reproducibility. We report, herein, the fabrication of Ag-rich Au-Ag alloy nanowires using template-based electrochemical deposition. This specific technique was selected owing to its properties of facile synthesis, achievable NW array format, and scalable process. Dealloying process was applied, though mild acid etching was performed to slightly generate roughened surface of alloy NWs without major loss of Ag composition. The novel controlled dealloying process proposed here not only offered an improved SERS signal from roughened surface, but surprisingly high stability can also be obtained without diminishing the initial SERS signal, in contrast to previously reported procedures of protective layer coating.

To frame the key accomplishments, this paper addresses (1) the fabrication process and physical/chemical characteristics of the porous alloy nanowires array based on SEM, TEM, EDX analysis, and (2) the performance of SERS substrate with respect to sensitivity, stability, uniformity and reproducibility.

## Results and Discussion

### Fabrication and characterizations of porous gold-silver alloy nanowires array

**Figure 1:**
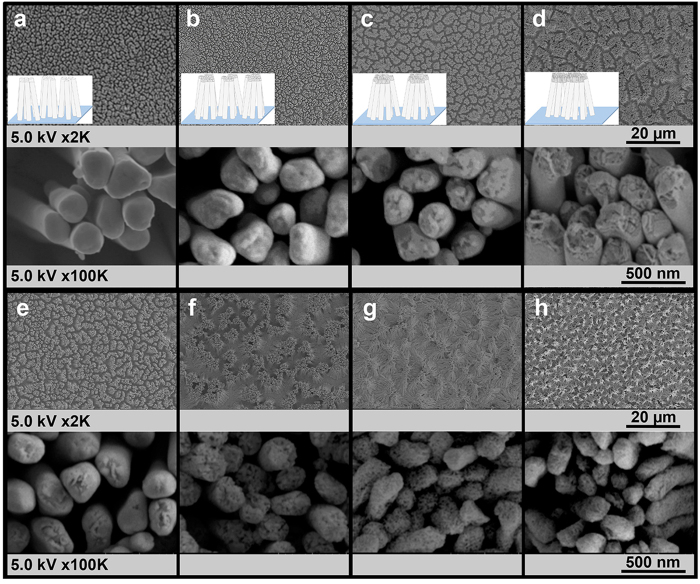
SEM images of (**a**) the AuAg-NWs array and the AuAg-NWs array etched with various etching conditions (nitric acid concentration-etching time); (**b**) 15%–1 min, (**c**) 15%–3 min, (**d**) 15%–5 min, (**e**) 20%–1 min, (**f**) 20%–3 min, (**g**) 20%–5 min, and (**h**) 30%–1 min. All images were taken at 2 K (above row) and at 100 K (bottom row) magnifications. Schematic representation of NWs array formation (**a**–**d**) was shown as an inset.

**Figure 2:**
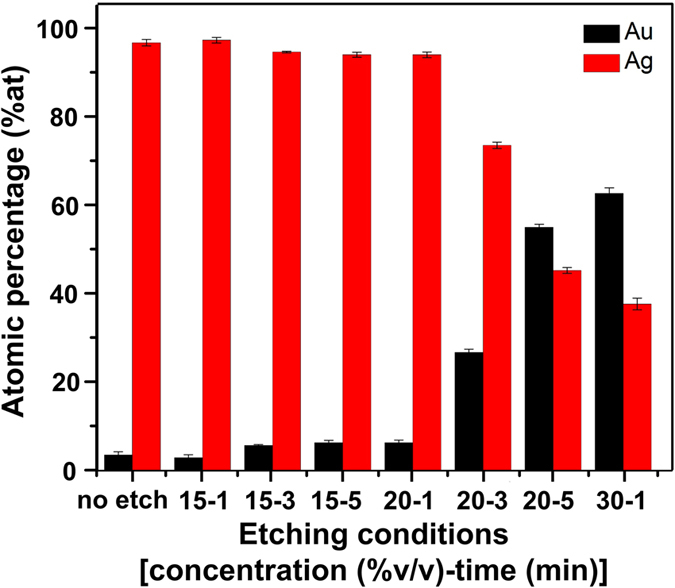
Bar graphs of averaged Au and Ag contents (atomic percentage, % at) of the NWs array as a function of the different etching conditions denoted as HNO_3_ concentration (%v/v)-time (min): (1) no etch, (2) 15-1, (3) 15-3, (4) 15-5, (5) 20-1, (6) 20-3, (7) 20-5, and (8) 30-1. Error bars represent standard deviation (SD) of measurements on five different areas of the NWs array.

It has been widely recognized that the use of template-based electrochemical deposition technique can be used to produce nanowires with controlled diameter, length, and elemental composition. Anodized aluminum oxide (AAO) membrane is the most commonly used template for NW fabrication due to its controllable pore size and high-density array in hexagonally close pack arrangement. Generally, the gold-silver alloy nanowires (Au-Ag alloy NWs) were fabricated within the membrane pores and released from AAO membrane to obtain the free-standing nanowire before sequentially dealloyed via acid etching to create the porous nanowires structure. Nitric acid can selectively dissolve Ag from Au-Ag alloy. It is understood that the porous-morphology evolution of the dealloyed nanowires directly relates to both nitric acid concentrations and etching time. Therefore, the morphology of porous Au-Ag alloy nanowires may be controlled by compensation between nitric acid concentration and etching time. The resulting NW structures are denoted here after as p-AuAg-NWs. We first investigated the possibility of using typical acid etching conditions of 30–35%v/v nitric acid solution for 15 min^20^. Unfortunately, the harsh acid conditions led to more than 95% release of NWs from the array and the substrate was not usable for further SERS analysis. Since there is no prior report on the morphological evolution of porous nanowires using mild etching conditions, 3 sets of experiments were performed in this study using nitric acid concentration of 15, 20, and 30%v/v and etching time of 1, 3, and 5 min. SEM images and EDX analysis were obtained from p-AuAg-NWs array as demonstrated in Figs 1 and 2, respectively. Similar results of an almost complete removal of NWs from the array were noticed when using 30%v/v nitric acid concentration and etching time of 3 and 5 min (data not shown). These indicate that such conditions are not suitable for p-AuAg-NWs array fabrication and, thus were excluded from further experiments. Considering SEM images of p-AuAg-NWs array obtained from other acid etching conditions, the etching of Au-Ag alloy NWs using high concentrations of nitric acid (20%v/v for 3, 5 min and 30%v/v for 1 min, Fig. 1f–h) was capable of rapid creation of the porous structure throughout the entire NWs segment. Whereas, the porous structure of the NWs were slightly developed as a function of time when etching with mild acid conditions (15%v/v for 1, 3, and 5 min and 20%v/v for 1 min, Fig. 1b–e). According to the low magnification images illustrated in Fig. 1, it can be simply noticed that the formation of leaning structure within the NW array predominantly occurred with the highly porous NW structures (20%v/v for 3, 5 min and 30%v/v for 1 min) due to their flexibility. This leaning effect is generally caused by the surface tension when a droplet of solvent (in this case water) evaporated from the substrate. However, the detachment of highly porous structures from the substrate also concurrently happened leading to low coverage of NWs array and hence low uniformity of SERS substrate. The corresponding elemental analysis was also demonstrated in Fig. 2. Porosity of the NWs apparently related to the atomic percentage (% at) of Au and Ag of the NWs array. Initially, silver was the major composition of Au-Ag alloy NWs with 96% at. As expected, the amount of silver continuously decreased, inversely with the amount of gold, upon increasing concentration of nitric acid or extension of etching time. Interestingly, a mild acid concentration of 15%v/v slightly affected the Au:Ag atomic ratio. A small decrease of Ag composition from 96% to 94% was measured for as-prepared Au-Ag alloy NW array and the array that was allowed to submerge in acid solution for 5 min, respectively. On the contrary, the characteristics of NWs surfaces as shown in SEM images (Fig. 1, the high magnification images) were significantly changed with higher surface roughness at longer etching time indicating a selective surface removal of silver. Great coverage of NW arrays was successfully achieved at these particular mild acid etching conditions (15%v/v). Moreover, larger aggregate size causing by the leaning effect was observed upon increasing etching time. These self-assembled hot spots generated from tip-to-tip aggregation are preferable owing to a large number of electromagnetic enhancements.

Also shown in Fig. 2 is a drastic decline of Ag composition (~95% to 37%) when using strong acid etching conditions (20%v/v for 3, 5 min and 30%v/v for 1 min), which have been considered too severe for formation of a uniform p-AuAg-NWs array. These compositional changes are strongly supported by porous NWs structures shown in the correlated SEM images (Fig. 1f–h). As mentioned above regarding ideal SERS characteristics, uniformity is one of the important parameters. Therefore, these results indicate that the integration of a simple and large area NWs array fabrication with the controlled dealloying process is a key success to create uniform SERS substrate. Furthermore, the etching time of 1–5 min at a suitable acid concentration (15%v/v) reported here is an essential tool for achieving a tunable surface roughness while maintaining the NWs array format, which can be further utilized as SERS substrate.

### Performance testing of the p-AuAg-NWs array as SERS substrates

**Figure 3:**
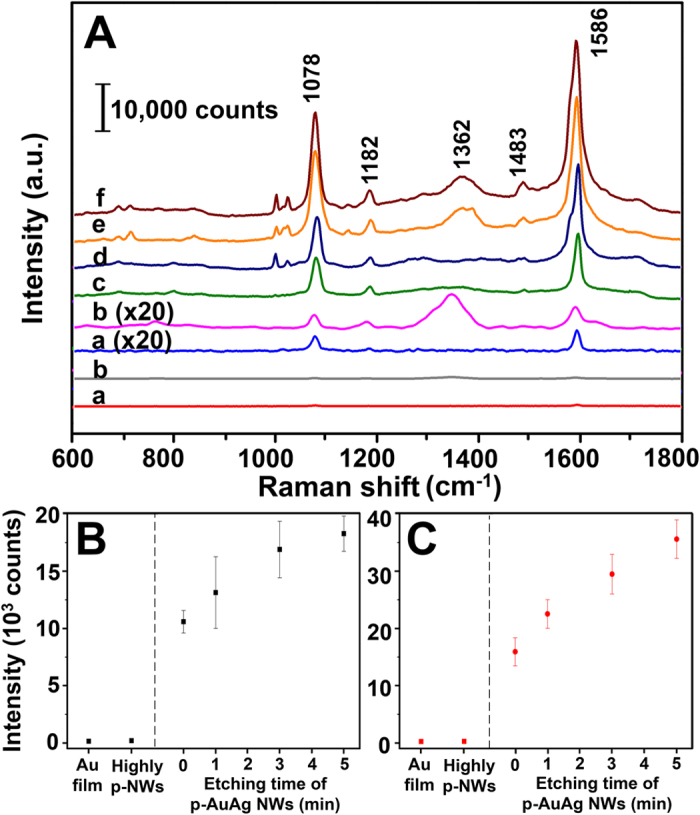
(**A**) Representative Raman spectra of 4-MBA on gold film (a), highly porous nanowires (20%v/v HNO_3_ etching for 5 min) (b), and the SERS substrates of p-AuAg-NWs array prepared by etching with 15%v/v HNO_3_ at various etching time of 0 (c), 1 (d), 3 (e), and 5 (f) min, respectively. The corresponding average SERS intensities were determined at (**B**) 1078 cm^−1^ and (**C**) 1586 cm^−1^. All signals were detected using 4-MBA (1 mM) as sample. Error bars represent standard deviation (SD) of measurements on ten different areas of the NWs array (n = 10).

The structure of p-AuAg-NWs array is considered to be beneficial for SERS applications because an increase of surface roughness provides higher surface area for SERS-active molecule adsorption. Since various structures of p-AuAg-NWs arrays were fabricated in this work, SERS performance of the as-prepared substrates was investigated using 4-mercaptobenzoic acid (4-MBA) as Raman-active probe molecule. As demonstrated in Fig. 3, SERS substrates prepared from solid AuAg NWs, p-AuAg-NWs etched with 15%v/v HNO_3_ at various times (1, 3, and 5 min), 20%v/v HNO_3_ for 5 min, and gold film were all tested using the same conditions of Raman measurement. Figure 3A(a–f) presents SERS spectra of 4-MBA on each substrate. The pronounced peaks of 4-MBA are consistent with previous reports^4^,^25^,^26^. The ring breathing modes ν(CC), which are the characteristic peaks of 4-MBA, appeared for all SERS substrates at Raman shift of 1076 and 1586 cm^−1^. While other less intense modes, including δ(CH), ν_s_ (COO^−^), and ν(CC)+δ(CH) combination modes were easily noticeable with the higher SERS response at ~1182, 1362, 1483 cm^−1^, respectively.

To compare the SERS performance of each sample, average signal intensities of both characteristic peaks (1076 and 1586 cm^−1^) were calculated and presented in Fig. 3B and C, respectively. As expected, SERS signals of both characteristic peaks increased as a function of etching time. Significant enhancement was observed from 0 to 3 min etching, while a slight increase was detected for an extended time of 5 min. These results were strongly supported by SEM/EDX analysis discussed in the previous section. It indicated that the use of longer etching time provided a higher surface area while keeping the relatively constant Au and Ag compositions, generating amplification of SERS hot spots. Therefore, such simple approach of controlled dealloying could successfully offer the p-AuAg-NWs array with enhanced SERS activity. However, the structure of nanowires can be affected when using too severe conditions for dealloying. To demonstrate this complication, the p-AuAg-NWs array (etching condition of 20%v/v, 5 min) was also prepared for SERS measurement. The p-AuAg-NWs array was selected as a representative of etching condition, which results in obviously high porosity and considerable change of Ag composition. According to the results shown in Fig. 3, extremely low signals were collected from both gold film and highly p-AuAg-NWs array substrates. At least 50 times of magnification is required to approximately reach the SERS signal obtained from the solid AuAg NWs. Furthermore, an exceedingly higher SERS signal (88–150 fold) was obtained from the p-AuAg-NWs with controlled etching conditions of 15%v/v for 5 min. Consequently, this particular SERS substrate was selected for further performance testing due to the highest SERS signal with the enhancement factor (EF) value of 5.41 × 10^6^ (see Supplemental Information for further details of EF calculation).

#### Sensitivity of SERS substrate

**Figure 4:**
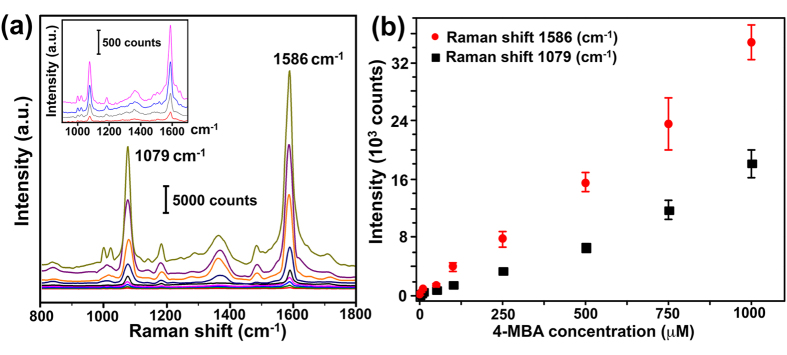
Sensitivity tests of the p-AuAg-NWs array SERS substrate produced from 5 min of 15% HNO_3_ etching. (**a**) SERS spectra of 10^−6^ M–10^−3^ M 4-MBA on the fabricated porous alloy nanowires, (**b**) calibration curves of average SERS signal intensities of the characteristic peaks at 1079 and 1586 cm^−1^ vs. 4-MBA concentrations. Error bars represents standard deviation (SD) of measurements on ten different areas of the NWs array (n = 10).

SERS spectra of 4-MBA at different concentrations on the selected SERS substrate were collected as shown in Fig. 4a in order to test its detection sensitivity. According to the graph, the spectral features of 4-MBA signals were detectable in a wide range of micromolar to millimolar concentration. Even at the low concentration, as low as 5 × 10^−6^ M, the 4-MBA signal was still distinctive (shown in Fig. 4a, inset). The intensities of the characteristic peaks of 4-MBA were measured to obtain the calibration curves as given in Fig. 4b. The relationships between SERS signals and 4-MBA at various concentrations were linear in the range of 10^−6^ to 10^−3^ M with regression (r^2^) of 0.988 and 0.995 for the intensity at Raman shift of 1079 and 1586 cm^−1^, respectively. It confirmed that the fabricated p-AuAg-NWs array is suitable for application as a highly sensitive SERS substrate.

#### Stability of SERS substrate

**Figure 5:**
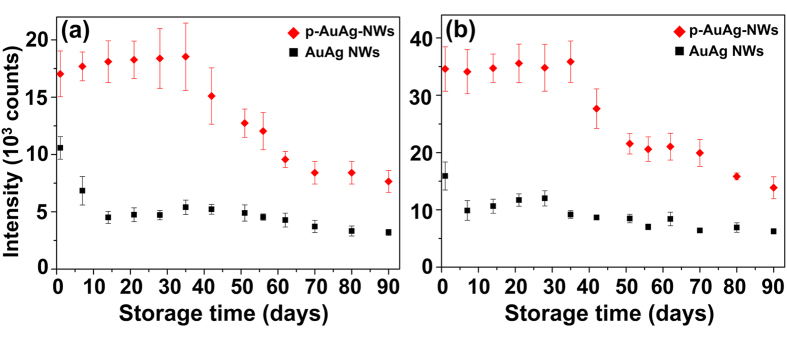
Stability of the SERS substrates prepared from Au-Ag NWs array with (red) and without (black) controlled dealloying (15% HNO_3_ for 5 min). Average intensities of 4-MBA peaks at (**a**) 1078 cm^−1^ (**b**) 1586 cm^−1^ vs. days after fabrication. All signals were measured using 4-MBA (1 mM) as a Raman probe. Error bars represent standard deviation of ten measurements (n = 10).

As emphasized above, one of the ultimate goals for development of SERS substrates is obtaining high stability while maintaining its sensitivity. However, the previously reported techniques such as the use of metal oxide or inert metal coating layers only offer stability improvement at the sacrifice of SERS sensitivity^11^,^22^,^23^,^24^,^27^,^28^,^29^,^30^,^31^,^32^,^33^. In order to truly examine the value of the proposed technique towards SERS substrate fabrication, the stability of both p-AuAg-NWs array (Fig. 5, red dot plots) and AuAg NWs array (Fig. 5, black dot plots) were simultaneously monitored for a total period of 90 days. The as-prepared SERS substrates were kept at room temperature without special storage conditions. Raman signals of 4-MBA on the SERS substrates were measured as a function of time after synthesis and average peak intensities are shown in Fig. 5.

It can clearly be seen in Fig. 5 (a, b, red dot plots) that both characteristic Raman shifts (1078 and 1586 cm^−1^) of the p-AuAg-NWs array substrates provide the highly stable SERS signal of 4-MBA for at least 35 days (100% of the original signal) before a gradual decrease. After 90^th^ day, the 4-MBA signals measured from the p-AuAg-NWs array were calculated to be approximately 45 and 40% of the initial signal at the respective Raman shifts of 1078 and 1586 cm^−1^. Whereas, the drastic decrease in SERS activity (as high as 38% signal reduction) was obtained from the solid AuAg-NWs array substrate within the first 7 days. After that, the SERS activity continuously reduced over storage time of 90 days and only maintained 30 and 40% of the initial signals. This is considered a very interesting finding due to the fact that the p-AuAg-NWs array substrate is not only offered higher sensitivity with respect to solid AuAg-NWs array substrate (Fig. 3), but also exhibited high stability over 3 months of storage time (Fig. 5). We have considered the other techniques commonly applied for stability improvement of SERS substrate. For examples, atomic layer deposition (ALD) was used to coat Ag NWs array with an ultrathin TiO_2_^22^ or Al_2_O_3_^11^ coating layer, a highly stable signal can be achieved for approximately 50 days in the air for both materials. Nevertheless, it is important to note that by coating Ag NWs array with TiO_2_ or Al_2_O_3_ using even single ALD cycle (<1 nm thick), normalized Raman intensity of the coated substrate was calculated to be only 60% of the uncoated structure and slowly decrease to approximately 40% of its original signal after 50 days. Unlike our controlled dealloying process, Raman intensities for the 1078 and 1586 cm^−1^ Raman peaks were significantly improved by 72 and 124% and also maintained 100% of initial signal for at least 35 days. Several other materials such as graphene^24^,^29^, silica^31^, Au^23^, polyvinyl alcohol (PVA)^33^ were employed as coating layer for an excellent stability improvement. Unfortunately, all of them also exhibited the initial reduction of SERS signal.

**Figure 6:**
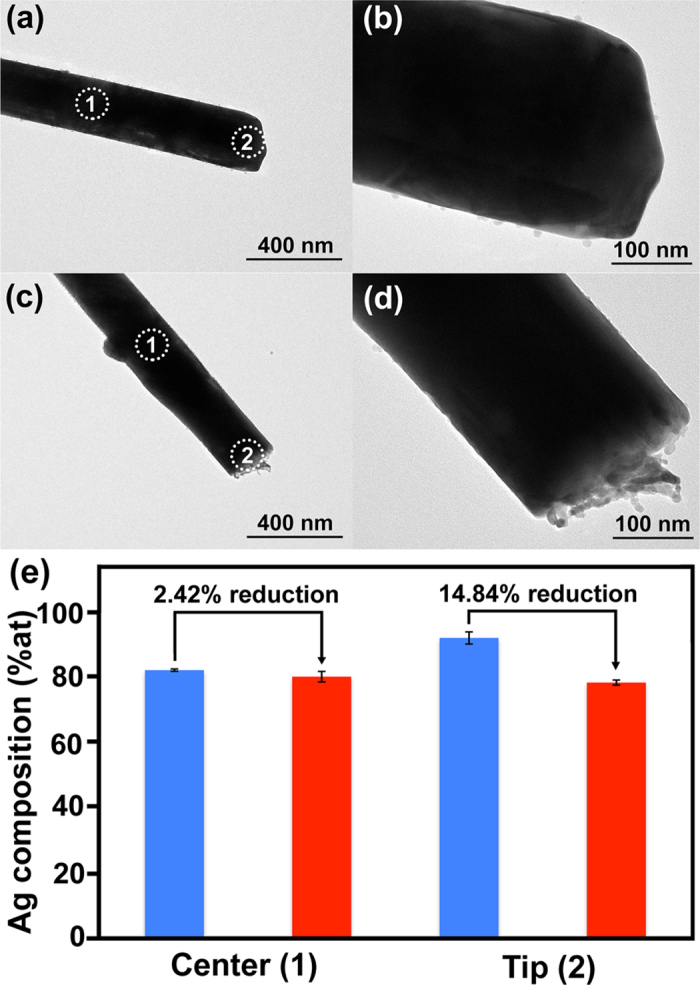
Low (**a**,**c**) and high (**b**,**d**) magnification TEM images of single solid AuAg NW (**a**,**b**) and p-AuAg-NW (**c**,**d**) removed from the SERS substrate without and with acid etching (15%v/v HNO_3_ for 5 min), respectively. Bar graph (**e**) demonstrated atomic percentage (% at) of Ag at the center (1) and tip (2) of NW, which were determined by EDX analysis. Blue and red bars exhibit the average Ag compositions before and after etching, respectively. Error bars represent standard deviation of five measurements (n = 5).

To further investigate the structural characteristics that are responsible for highly stable and sensitive SERS signal, TEM/EDX analysis was performed on the single NW structure released from AuAg-NWs array and p-AuAg-NWs array (15%v/v HNO_3_ etching for 5 min). TEM images of the nanowires are shown in Fig. 6(a–d). In general, the dealloying process starts when the nanowire surface is exposed to the acid etching solution. Silver atoms within the AuAg alloy structure are preferentially dissolved generating non-coordinated Au atoms. These reactive Au atoms, called Au adatoms, are then reorganized themselves by diffusing and agglomerating into larger gold islands/ligaments. The continuation of dealloying process occurs inwards and eventually creates highly porous structure^20^,^34^,^35^,^36^. According to the high and low magnification TEM images shown in Fig. 6, a surface morphology of the AuAg-NW (a, b) prior to etching appeared smooth, while the p-AuAg-NW (c, d) demonstrated an evidence of island formation (roughened surface) at the tip of NW. The roughened surface appeared particularly at the tip of NW suggested that the acid etching of AuAg-NW array initially occurred from top surface of the array and slowly moved downwards. This etching process differs from the etching of free-standing NWs, which results in non-selective area etching. Typically, a large volume (1 mL) of etching solution is used to dealloy NWs that are suspended in solution. Thus, all NWs surface is exposed to the etching solution and a dealloying process occurs simultaneously at the tip and sidewall of NWs. In contrast, a small amount (50 μL) of etching solution was used in this study and it was particularly applied to a leaning NWs array. This structure limits an exposure of the NWs sidewall to the etching solution and allows the selective etching at the tips of NWs. In addition, the observed change well corresponds to an early phase of the evolution of porous gold nanostructures described above. The island formation suggested that small amount of Ag atoms were removed from the surface resulting in porous alloy layer with higher Au composition. TEM-EDX analyses were performed on both AuAg-NWs and p-AuAg-NWs. Atomic percentage (%at) of Ag at the tip and center of NWs are presented in Fig. 6e. The results confirmed that there is a reduction of Ag composition at the center and tip of nanowires after acid etching at 2.42 and 14.84%, respectively. Selective etching of NW tip offers an exceptional advantage of improved stability and sensitivity. An increase of Au composition only on the NW tips (the top surface of NW array) without dramatic change of NW array composition is potentially accounted for the high stability (less oxidative surface). In the meantime, Raman hot spots were concurrently generated between the roughened surfaces, which provide higher sensitivity (amplified hot spots).

#### Uniformity and reproducibility of SERS substrate

**Figure 7:**
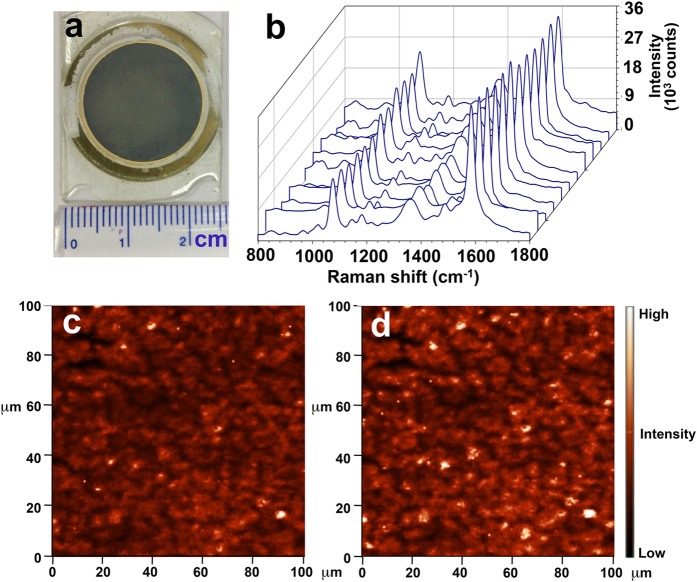
Uniformity of SERS substrate determined at macroscopic and microscopic areas. For a large area, a photograph of SERS substrate containing p-AuAg-NWs array (**a**) and SERS spectra of 4-MBA randomly collected from 12 positions on the SERS substrate (3.14 cm^2^) (**b**). For a small area, Raman images represent signal intensities of 4-MBA at the Raman shifts of (**a**) 1078 cm^−1^ and (**b**) 1586 cm^−1^ within a mapping area of 100 × 100 μm^2^.

One of the major challenges for an application of SERS substrate is the capability of producing uniform and reproducible SERS signals. Uniformity of SERS substrate can be obtained from homogenous distribution of plasmonic nanostructures; hence, reliable SERS signal for quantitative detection. Reproducibility is mainly accomplished via a well-controlled nanostructure fabrication resulting in similar batch-to-batch synthesis. In this work, the uniformity of p-AuAg-NWs SERS substrate was investigated for both macroscopic and microscopic area. For the large-area uniformity, SERS signals were randomly collected from 12 positions on the p-AuAg-NWs SERS substrate within a circular area of 2-cm diameter as shown in Fig. 7(a,b). SERS intensities of the characteristic peaks were measured. Average and percentage of relative standard deviation (%RSD) were determined in order to investigate the spot-to-spot uniformity of the whole area (3.14 cm^2^). According to the results, the average intensities at 1078 and 1586 cm^−1^ were 15414 ± 1488 counts (%RSD = 9.66, n = 12) and 35872 ± 4361 counts (%RSD = 12.16, n = 12), respectively. This value is acceptable considering other previously reported values of 8–12.6% for centimeter scale SERS substrates^9^,^37^,^38^,^39^. Another experiment involved Raman mapping was performed to determine the uniformity within a microscopic area. SERS spectra of 16,834 individual spots were collected from the square area (100 × 100 μm^2^) on the substrate with 0.5 s integration time. The resulting Raman map comprising 16,384 spectra was analyzed. The relative intensities of 4-MBA at Raman shifts of 1078 and 1586 cm^−1^ are shown in Fig. 7(c,d), respectively. SERS signal intensities at both Raman shift are quite uniform with normal Gaussian distribution (see Fig. S1 in Supplemental Information). The uniformity was estimated from %RSD, which were 2.35 and 2.96 for the signal intensities at Raman shift of 1078 and 1586 cm^−1^, respectively. The acceptable RSD values indicate the excellent uniformity of the reported SERS substrate, both in macro- and micro-scales (the whole membrane area and 100 × 100 μm^2^ area, respectively). These collective data confirmed that the simple technique of controlled etching of NWs array reported here is suitable for fabrication of SERS substrate at the centimeter scale area with micrometer scale precision.

To demonstrate batch-to-batch variation of the reported procedure, 5 different SERS substrates of the p-AuAg-NWs array were fabricated. Average Raman intensities at 1078 and 1586 cm^−1^ Raman shift were calculated from 10 spectra and shown in Fig. S2. Relative standard deviations (RSD) of the 5 different samples were determined to be 10.9% and 3.3% for the corresponding Raman shifts demonstrating acceptable inter-variation.

## Conclusions

The highly stable and sensitive SERS substrate can be accomplished using a simple approach of template-based electrodeposition followed by controlled dealloying process. It was found that the controlling of etching conditions (i.e. etching time or nitric acid concentration) dramatically affects SERS activity. Under the optimized etching condition, the resulting structure of p-AuAg-NWs array exhibited the leaning NWs formation. Amplification of SERS hotspots was also observed via the formation of porous structures on the NW tip. This leads to enhance SERS activity with EF of 5.41 × 10^6^. Moreover, this particular structure offers a great long-term stability without the addition of protective coating layer. One hundred percent of the original SERS signal was maintained for at least 35 days and the signal can be detected after 3 months of storage time. The uniformity of the whole SERS substrate was considerably acceptable with an RSD less than 12%. Small variation of less than 3% RSD was obtained within a microscopic area of 100 × 100 μm^2^. The SERS substrates also exhibited small variation of batch-to-batch synthesis. According to the full consideration of SERS properties (sensitivity, stability, uniformity and reproducibility), the controlled dealloying process reported herein can provide a simple route to create an excellent SERS substrate for further applications in bio- and chemical sensors.

## Materials and Methods

### Chemicals and materials

Anodic aluminum oxide (AAO) membranes with pore size of 0.2 μm and thickness of 60 μm were obtained from Whatman Corporation (Anodisc 25, catalog No. 6809-6022; UK). Gold sputtering target was from Quorum Technologies Ltd., USA. An electrochemical cell were custom made with Teflon cylinder (1.25 cm inner diameter) and stainless steel plate (W = 5 cm, L = 5 cm, H = 0.5 cm). The reference and counter electrodes were Ag/AgCl and 0.5 mm-diameter platinum wire, respectively.

All chemicals were purchased from a commercial source and used without further purification. Deionized (DI) water used in all experiments was obtained from Thermo scientific water purification system with an electronic resistance 18.2 MΩ. Gold plating solution (Orotemp 24 RTU RACK) and silver plating solution (1025 RTU @4.5 troy/gallon) were purchased from Technic (Cranston, RI, USA). Alloy plating solution was prepared by mixing gold and silver plating solutions with volume ratio of 70:30. Nitric acid for etching process was diluted from 65% nitric acid obtained from CARLO ERBA, France. A Raman probe, 4-mercaptobenzoic acid (catalog no. 706329), was purchased from Sigma-Aldrich, USA. Adhesive glue used for substrate preparation is cyanoacrylate adhesive (catalog no. Z105902) from Sigma Aldrich, USA.

### Instrumentation

All electrodeposition experiments were performed with a CHI 600D potentiostat (CH Instruments, Austin, TX). Platinum wire and Ag/AgCl served as the counter and reference electrodes, respectively. Scanning electron microscopy (SEM) imaging and an elemental analysis of NWs array were carried out using an electron microscope (SU8030 Hitachi, Hillsboro, OR), equipped with an energy dispersive X-ray analyzer (Amatek Inc., Mahwah, NJ) under an accelerating voltage of 10 kV. Transmission electron microscopy (TEM) images were captured at operating voltage of 160 kV and localized elemental compositions were performed on single NW using TEM-EDS detector (JEOL2010, USA). Raman spectra and mapping were measured using AFM-correlated confocal Raman spectrometer (NTEGRA Spectra, NT-MDT, Moscow, Russia).

### Fabrication of p-AuAg-NWs array and SERS substrate preparation and characterization

Gold-silver alloy nanowires were fabricated using AAO membrane as a template during electrodeposition. Before nanowire synthesis, a thin gold film (approximately 200 nm thick) was firstly coated on the branch side of AAO membrane via DC sputtering in order to obtain a conductive film. The gold-coated membrane was then used as a working electrode. Prior to assembly in the custom-made electrochemical cell, the membrane was filtered with ultra-purified water to increase hydrophilicity within the pores. For the growth of alloy nanowires, a mixture of gold and silver-plating solutions at the volume ratio of 70:30 was electroplated into the membrane’s pores as shown in Fig. S3. The designated charge of 5 coulombs (C) was allowed for the nanowires growth, which was carried out at a potential of −0.9 V. Finally, the membrane was released from the electrochemical cell and rinsed with ultra-purified water for residual removal.

For SERS substrate preparation, the membrane filled with the alloy nanowires was left until dried. Then, an adhesive glue was used to adhere the conductive gold film side of the membrane onto a glass slide. It is important to note that a smooth adhesive layer was required to create a homogenous SERS substrate. After an hour of drying process, the alumina template was removed by submerging the glass slide with the membrane in 3 M NaOH for 30 min. Strong basic solution of NaOH selectively dissolved the alumina template resulting in an alloy nanowires array. The nanowires array was gently rinsed with ultra-purified water for complete removal of the residue. To prepare porous alloy nanowires array, acid-etching process was applied to the alloy nanowires array described above. Typically, a 50 μL of 15% HNO_3_ was dropped onto the nanowires array surface forming a hemisphere drop covering the whole area (3.14 cm^2^). The dealloying process was allowed to continue for 1–5 min prior to immediate rinsing with DI water. Higher concentration (20 and 30%) of nitric acid solution was also used to prepare a highly porous structure. The arrangement of nanowires arrays and their elemental compositions were characterized using SEM and TEM coupled with EDX analysis. SEM/EDX analysis was used to characterize NWs array, while TEM/EDX analysis was applied for a single NW measurement. For SEM/EDX analysis, 5 measurements were collected from different scan areas (approximately 3000 μm^2^ at 2000X agnification) of the nanowire array. For TEM/EDX analysis, NWs were removed from the array with the aid of slight sonication and dropped onto TEM grid. Five different nanowires were randomly selected and characterized particularly at the tip and the center of nanowire length.

### Raman spectroscopy measurement

A Raman probe, 4-mercaptobenzoic acid (4-MBA), was used to evaluate the SERS performance of the as-prepared substrate. Various concentrations of 4-MBA were prepared in ethanol at 1.0, 0.5, 0.1, 0.05, 0.01, 0.005 and 0.001 mM. A 5 μL of each solution was dropped on the porous alloy nanowires array and dried out. SERS spectra of the samples were collected using a 632.8 nm laser excitation at 100 μW power (ND filter 1, exposure time 1 s, total collection time of 60 s). For other SERS measurements including a comparison of porous and non-porous nanowires array, stability test, and uniformity and reproducibility test, a solution of 1 mM 4-MBA was used. SERS spectra of the samples were collected as described above with an exception of Raman mapping which an exposure time for each spot was set to 0.5 s.

### SERS enhancement factor calculation

The enhancement factor (EF) of p-AuAg-NWs SERS substrate was calculated using the equation EF = (*I*_*SERS*_/*N*_*SERS*_)/(*I*_*bulk*_/*N*_*bulk*_), where *I*_*SERS*_ and *I*_*bulk*_ are the intensities of 4-MBA at 1586 cm^−1^ Raman shift of the SERS and normal Raman spectra, respectively. *N*_*SERS*_ is the number of 4-MBA molecules excited on SERS substrate. *N*_*bulk*_ is the number of molecules excited within a solid 4-MBA sample. The SERS and normal Raman spectra were measured using the same Raman conditions (a 632.8 nm laser excitation at 3 mW power, ND filter 0, exposure time 1 s, total collection time of 60 s). Detailed calculation is given in the Supplemental Information.

## Additional Information

**How to cite this article**: Wiriyakun, N. *et al*. Site-Selective Controlled Dealloying Process of Gold-Silver Nanowire Array: a Simple Approach towards Long-Term Stability and Sensitivity Improvement of SERS Substrate. *Sci. Rep.* **6**, 39115; doi: 10.1038/srep39115 (2016).

**Publisher's note:** Springer Nature remains neutral with regard to jurisdictional claims in published maps and institutional affiliations.

## Supplementary Material

Supplementary Information
