# Supplementary material for: Site-Selective Controlled Dealloying Process of Gold-Silver Nanowire Array: a Simple Approach towards Long-Term Stability and Sensitivity Improvement of SERS Substrate
Source: Sci Rep. 2016 Dec 13;6:39115. doi: 10.1038/srep39115 (PMC5153644; doi:10.1038/srep39115)
Supplement: Supplementary Information [file srep39115-s1.docx]

**Supplementary Information**

**Site-Selective Controlled Dealloying Process of Gold-Silver Nanowire Array: a Simple Approach towards Long-Term Stability and Sensitivity Improvement of SERS Substrate**

Natta Wiriyakun, Karuna Pankleaub, Suwimon Boonrungsiman, Rawiwan Laocharoensuk^*^

National Nanotechnology Center (NANOTEC), National Science and Technology Development Agency (NSTDA), Pathum Thani 12120, Thailand

*Corresponding author: rawiwan.lao@nanotec.or.th

**Enhancement Factor (EF) calculation**

The enhancement factor (EF) value of p-AuAg-NWs array SERS substrate (15%v/v HNO_3_, 5 min etching) was evaluated using 4-mercaptobenzoic acid (4-MBA) as a Raman probe. Intensities of characteristic peak at 1586 cm^-1^ Raman shift (ν(CC) ring breathing mode) were measured. Direct comparison of Raman spectra obtained from the SERS substrate and non-enhanced solid MBA was calculated according to the following formula:

*EF* = *(I_SERS_/N_SERS_)/(I_bulk_/N_bulk_)* ---------(Eqn. 1)

Where, *I_SERS_* and *I_bulk_* are the vibrational intensities of the 4-MBA molecules on the SERS substrate and solid MBA, respectively. *N_SERS_* and *N_bulk_* refer to the number of probe molecules under laser excitation of the SERS substrate and solid MBA, respectively.

In our experiment, a 5 µL of 1×10^-3^ M of 4-MBA (density of 1.346 g/cm^3^) was dropped on the SERS substrate. After evaporation a circular spot was formed covering an area of 1.77×10^-4^ m^2^. Assuming the 4-MBA molecules distributed evenly within the area (confirmed by unnoticeable film under the optical microscope), the average surface density of 4-MBA is 1.70 ×10^19^ molecules/m^2^. Since Raman data collection was operated at 632.8 nm using 40X objective lens (N.A. = 0.65) with a diameter of 1.2 µm spot size, the number of 4-MBA molecules on SERS substrate (*N_SERS_*) excited within the laser spot area can be calculated using Eqn. 2. The *N_SERS_* value of 1.92×10^7^ molecules was determined. For the solid MBA sample, the number of 4-MBA molecules was defined by the sampling volume, which takes into account the penetration depth of the laser beam (focal length = 14 µm). Therefore, the calculated number of 4-MBA molecules (*N_bulk_*) was 8.30×10^10^ molecules according to Eqn. 3. Based on the calculations in Eqn 1-3, *EF* from p-AuAg-NWs array substrate is determined to be 5.41 × 10^6^.

*N_SERS_* = 1.7 ×10^19^ (molecules/m^2^) × 1.13 ×10^-12^ (m^2^) = 1.92×10^7^ molecules------(Eqn 2)

*N_bulk_* = 1.346 (g/cm^3^) × 1.58×10^-17^ (m^3^) × 6.02×10^23^ = 8.30×10^10^ molecules------(Eqn 3)

154.19 (g/mol)

**
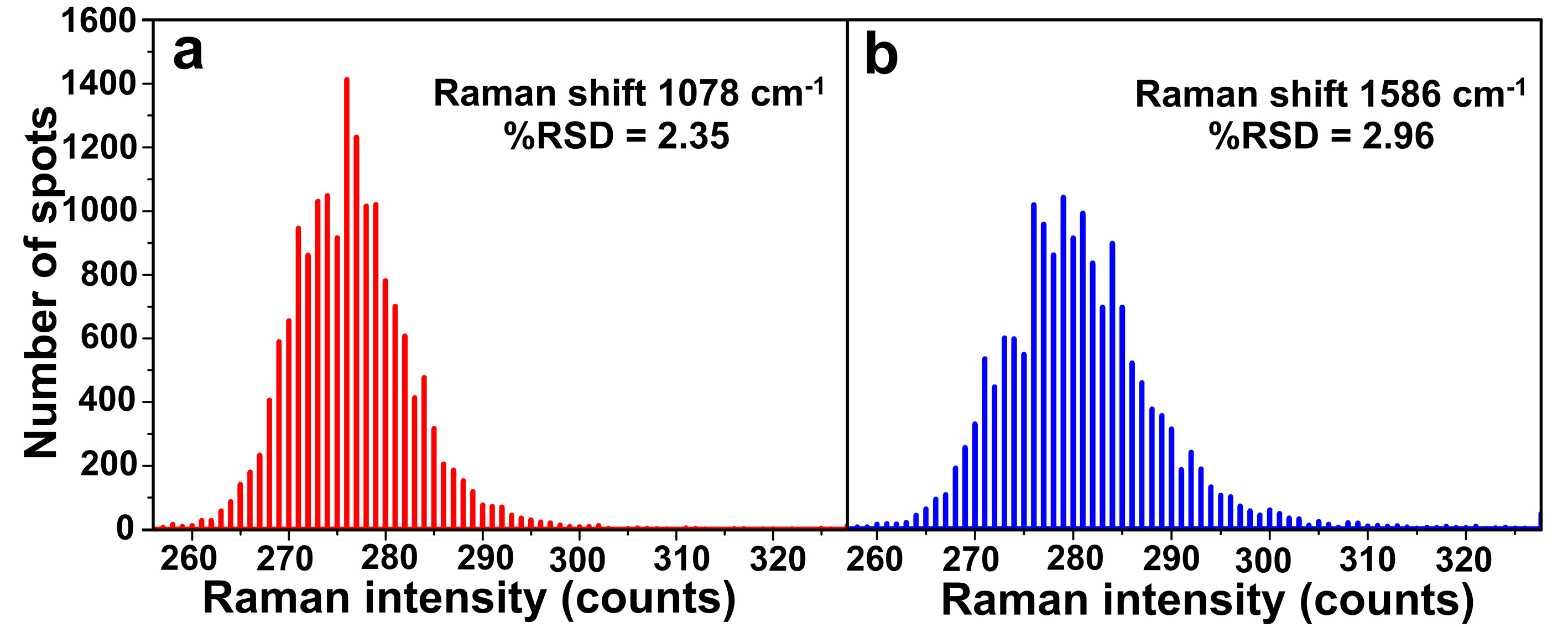
**

**Figure S1** Histograms of 4-MBA Raman intensities at (a) 1078 cm^-1^ and (b) 1586 cm^-1^ obtained from 16,384 spots of Raman mapping shown in Figure 7 (c-d). Relative standard deviation (RSD) of each characteristic peak was calculated to be 2.35 and 2.96%, respectively.


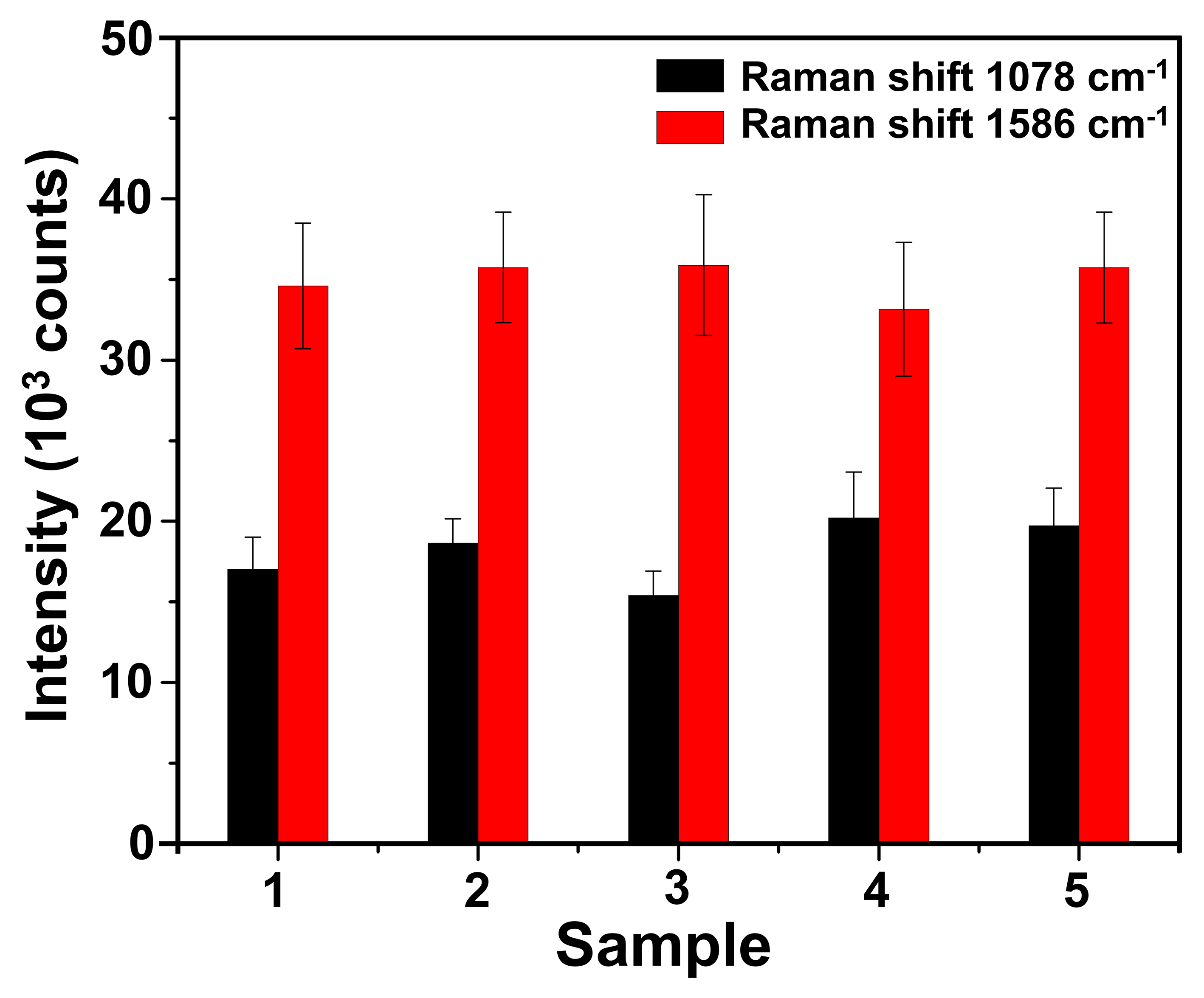


**Figure S2** Average intensities at 1078 and 1586 cm^-1^ Raman shifts measured from 5 different SERS substrates prepared by etching AuAg-NWs array with 15%v/v HNO_3_ for 5 min. Error bars represent standard deviation (SD) values of intra-variation within the sample (n =10).

**
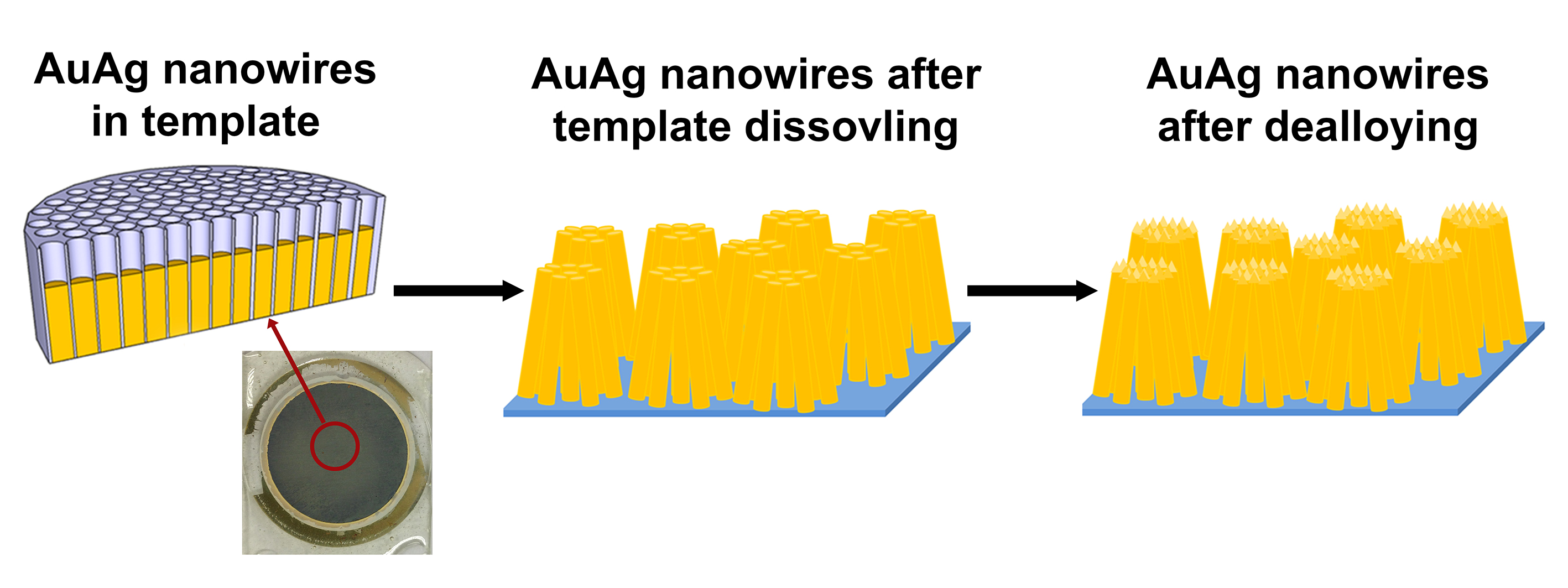
**

**Figure S3** Schematic representation of 3-step fabrication process of p-AuAg-NWs SERS substrate. The first step involved template-based electrodeposition of AuAg-NWs followed by the AuAg-NWs array formation via template removal. The final step for signal enhancement was done through silver dealloying using mild acid etching condition.
